# The Effect of Nitrogen Functional Groups on Pb^0^, PbO, and PbCl_2_ Adsorption over a Carbonaceous Surface

**DOI:** 10.3390/molecules29020511

**Published:** 2024-01-19

**Authors:** Liang Wang, Huaizhou Wen, Lei Guo, Ancheng Liang, Tingan Liu, Dongxu Zhao, Lu Dong

**Affiliations:** 1 China Power Hua Chuang (Suzhou) Electricity Technology Research Company Co., Ltd., Suzhou 215125, China; qj1124778917@163.com (L.W.); 15862301540@163.com (L.G.); franklta@163.com (T.L.); 2 Xi’an Thermal Power Research Institute Co., Ltd., Xi’an 740032, China; wenhuaizhou@tpri.com.cn; 3 Haikou China Power Environmental Protection Power Generation Co., Ltd., Haikou 570106, China; dlrobin@163.com (A.L.); 18789893587@163.com (D.Z.); 4 State Key Laboratory of Coal Combustion, School of Energy and Power Engineering, Huazhong University of Science and Technology, Wuhan 430074, China

**Keywords:** nitrogen functionalization, lead species, DFT calculations, adsorption, activated carbon

## Abstract

Lead (Pb) pollution, especially from the incineration of municipal solid waste (MSW), poses a significant threat to the environment. Among all the effective methods, activated carbon (AC) injection serves as an effective approach for lead removal from flue gas, while the modification of ACs emerges as a crucial pathway for enhancing Pb adsorption capacities. Density functional theory (DFT) is employed in this study to investigate the mechanisms underlying the enhanced adsorption of Pb species (Pb^0^, PbO, and PbCl_2_) on nitrogen-functionalized carbonaceous surfaces. The results show that nitrogen-containing groups substantially enhance lead adsorption capacity, with adsorption energies ranging from −526.18 to −288.31 kJ/mol on nitrogen-decorated carbonaceous surfaces, much higher than those on unmodified surfaces (−310.35 to −260.96 kJ/mol). Additionally, electrostatic potential and density-of-states analyses evidence that pyridinic nitrogen atoms remarkably expand charge distribution and strengthen orbital hybridization, thereby augmenting lead capture. This research elucidates the role of nitrogen-containing functional groups in lead adsorption, offering valuable insights for the development of highly efficient biomass-derived activated carbon sorbents for lead removal.

## 1. Introduction

With potent bio-toxicity and a propensity for bioaccumulation, lead (Pb) is categorized as a highly toxic heavy metal pollutant, and its contamination has widely emerged as a prominent global concern [1,2,3]. The annual generation of municipal solid waste (MSW) is approximately 1.3 million tons [4], and every kilogram of MSW contains approximately 109 milligrams of lead [5]; it is reported that the incineration of MSW has become a major source of lead pollution [6,7]. Although lead species exist at relatively low concentrations in flue gas (0.18–0.26 mg/m^3^) [8], it is crucial to eliminate even trace amounts before its emission to avoid hazardous environmental impacts.

Among the existing pollution control methods, sorbents injection serves as a viable approach to flue gas cleaning. In recent years, various solid sorbents, including metal–organic frameworks [9,10,11], metal oxides [12,13,14], and activated carbon [15,16,17], have been well explored. Among all the solid sorbents, MOF and metal oxides, despite their favorable adsorption capacities, face limitations in large-scale application due to their high production costs and limited stability [10,13]. Conversely, activated carbon (AC), especially that from biomass pyrolysis, has been recognized as an economical and effective approach for pollutant control in flue gases due to its robust adsorption capabilities and abundance [15]. However, the application of this technology is constrained by the limited adsorption capacity of activated carbon.

In general, the adsorption capacity of activated carbon is determined by its specific surface area and functional groups. Various methods have been employed to enhance the specific surface area of activated carbon, but further improvements in performance are restricted due to an upper limit in optimizing the pore structure [18]. Alternatively, the modification of the functional groups on activated carbon has emerged as a viable approach to enhancing its adsorption capacity [19,20]. Choi and Lee [20] utilized different chlorine-containing solutions to modify activated carbon for mercury adsorption and discovered that CuCl_2_-impregnated activated carbon exhibited the most rapid adsorption performance. In our previous research [21], the influence of phosphorous functional groups on arsenic adsorption was explored using experimental and density functional theory (DFT) methods, revealing that phosphorous functional groups significantly promote arsenic adsorption on carbonaceous surfaces. These findings collectively underscore the significance of functional group modification in increasing the adsorption capacity of activated carbon.

It is notable that nitrogen is a common element in biomass, migrating to gas, liquid, and solid products during pyrolysis, with amino, pyridine, and pyrrolic being the main forms of nitrogen in pyrolysis char. Additionally, conducting biomass pyrolysis in an NH_3_ atmosphere can increase the number of N-containing functional groups. Gao et al. [22] investigated the effect of N-doped functional groups on phenol adsorption by activated biochar and found that the adsorption capacities of different N-containing functional groups varied. Chen et al. [23] modified biomass-based materials and conducted mercury adsorption experiments, discovering that specific nitrogen-containing functionalities can greatly enhance adsorption capacity. It could be concluded that the addition of nitrogen-containing functional groups over a carbonaceous surface exerts a positive effect on pollutant adsorption. Additionally, numerous studies [24,25,26,27] have demonstrated the enhanced lead adsorption capabilities of nitrogen-doped and nitrogen-modified activated carbons through a serious of experimental studies, highlighting the pivotal role of nitrogen functional groups in improving the adsorption efficiency of activated carbon for lead ions, which needs to be further investigated. However, the effect mechanism of nitrogen-containing functional groups is still unclear. Quantum chemical calculation has been considered as a reliable tool for exploring the mechanism of adsorption reactions [28,29,30,31,32]. While extensive efforts have elucidated the fundamental mechanisms of lead removal by activated carbon, the specific reaction pathways involving lead species and nitrogen-decorated carbon surfaces remain insufficiently understood. Bridging this knowledge gap is imperative to enabling the rational design of superior adsorbents. Therefore, it is crucial to systematically probe the adsorption mechanisms of lead species on chemically active sites of carbonaceous surfaces and thoroughly examine the impacts of nitrogen functional groups.

In general, there are several kinds of lead species in flue gas, including elemental lead (Pb^0^), lead oxide (PbO), and lead dichloride (PbCl_2_) [2,33,34]. In this study, the carbonaceous surface structure with various nitrogen-containing functional groups was employed to explore the adsorption processes of Pb^0^, PbO, and PbCl_2_ via density functional theory (DFT) calculations [35]. In addition, wave function analyses [36], including Mayer bond order (MBO), electrostatic potential (ESP), and density-of-states (DOS), were conducted to shed light on the adsorption process. By delving into the interactions at the molecular level, this research not only elucidates the adsorption mechanisms, but also provides vital insights for developing efficient biomass-derived activated carbon sorbents optimized for lead removal.

## 2. Results

### 2.1. Pb^0^, PbO, and PbCl_2_ Adsorption over a Bare Carbonaceous Surface

**Figure 1:**
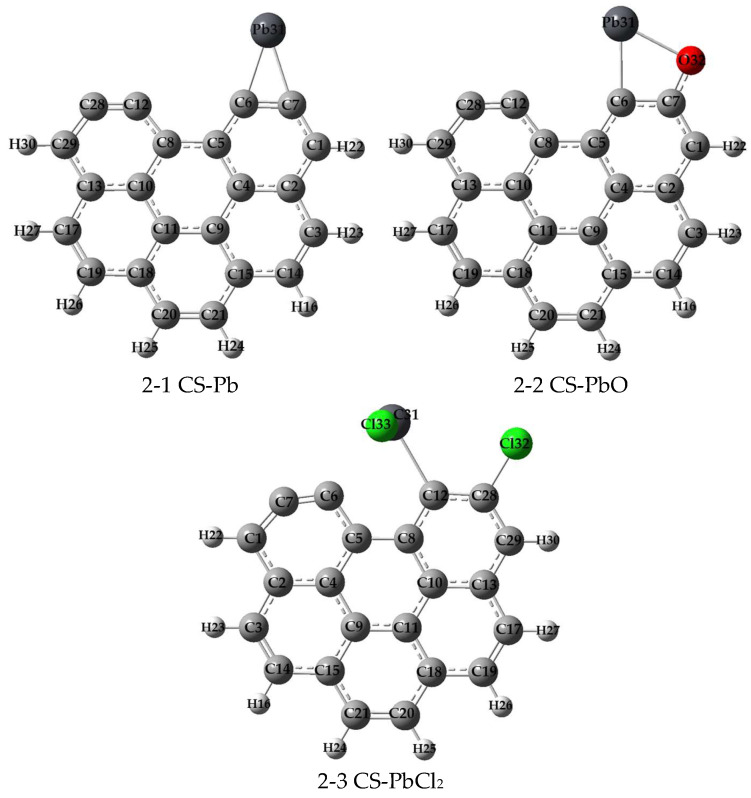
Structures of Pb^0^, PbO, and PbCl_2_ adsorption on CS (H, light gray; C, gray; O, red; Pb, black; and Cl, green).

In this study, to examine the adsorption characteristics of Pb^0^, PbO, and PbCl_2_ on carbonaceous materials, a model consisting of six armchair benzene rings was utilized as a representative carbonaceous surface structure [32]. To explore all the potential adsorption configurations, we considered and calculated every possible active site and the adsorption directions of lead species on the carbonaceous surface. By systematically examining these possibilities, we were able to identify the most stable adsorption structures for Pb^0^, PbO, and PbCl_2_, which are illustrated in Figure 1. Additionally, we quantified the adsorption energy associated with each configuration and documented several critical geometric parameters, as summarized in Table 1.

Figure 1 provides a visual representation of the adsorption configurations for Pb^0^, PbO, and PbCl_2_ on the pristine carbonaceous surface, denoted as structures 2-1, 2-2, and 2-3, respectively. Notably, our analysis revealed that the unsaturated carbon atoms at the edges of the carbonaceous material served as the active sites for adsorption in all cases. As shown in Table 1, the adsorption energies for these three configurations were found to be −310.35 kJ/mol for Pb^0^, −274.07 kJ/mol for PbO, and −260.96 kJ/mol for PbCl_2_. These negative values indicate a strong attractive interaction between the gases and the carbonaceous surface, signifying favorable adsorption. Furthermore, it is evident that the adsorption energy of structure 2-1 (Pb^0^) was the lowest among the three configurations, suggesting that Pb^0^ exhibited the highest affinity for adsorption on the carbonaceous surface compared to PbO and PbCl_2_. Notably, the optimized PbCl_2_ structure shows one Cl atom binding to a neighboring C atom rather than directly to Pb. This demonstrates the preferential interaction of one Cl atom with the carbon surface over the Pb. To gain deeper insights into the nature of the chemical bonds formed during the adsorption process, a Mayer bond order (MBO) analysis was employed, as listed in Table 1. The MBO analysis revealed that the bond order values were consistently close to 1.0 for all cases, indicating the formation of single bonds between the adsorbates and the carbonaceous surface.

### 2.2. Pb^0^, PbO, and PbCl_2_ Adsorption over a Carbonaceous Surface with Nitrogen Functional Groups

In adsorption studies, Gao et al. [37] extensively documented the preparation methods for nitrogen-doped biochar and underscored the pivotal role of nitrogen functional groups in enhancing the adsorption capabilities of carbonaceous materials. Their work revealed the presence of diverse nitrogen groups on the carbonaceous surface, including amino, pyridinic, and pyrrolic moieties, among others. These nitrogen groups have been recognized as crucial factors influencing the adsorption behavior of heavy metals, such as Pb^0^, PbO, and PbCl_2_.

**Figure 2:**
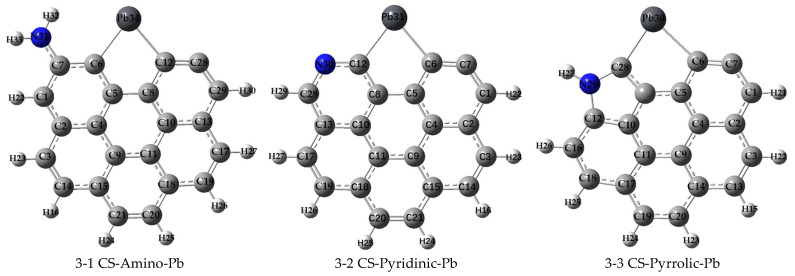

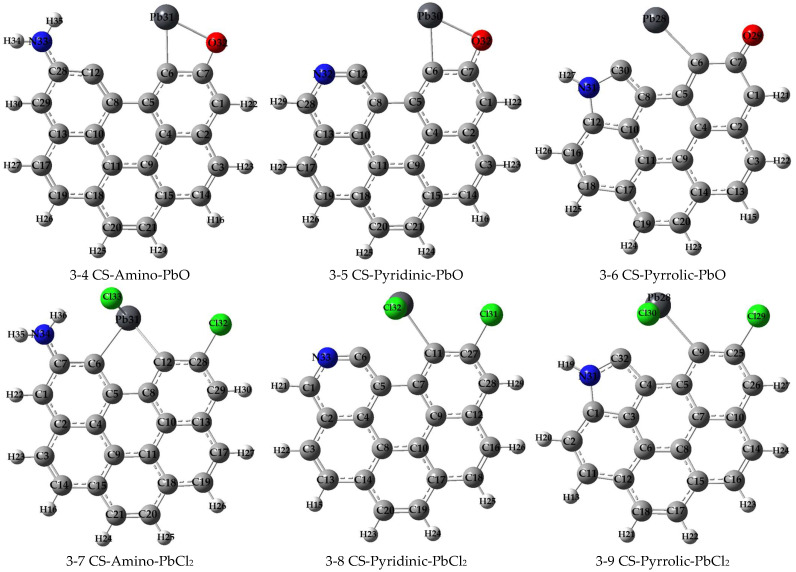
Structures of Pb^0^, PbO, and PbCl_2_ adsorption on CS with the N-containing functional groups (H, light gray; C, gray; O, red; Pb, black; Cl, green; and N, blue).

To comprehensively assess the effects of various nitrogen functional groups on the adsorption of Pb^0^, PbO, and PbCl_2_, distinct models with specific nitrogen functional groups were constructed and subjected to rigorous DFT calculations. This approach provided valuable insights into the adsorption characteristics arising from the nitrogen incorporation into the carbonaceous surface. After thoroughly optimizing these models, the final stable configurations were obtained, as shown in Figure 2. This illustrates the distinct adsorption behaviors of Pb^0^, PbO, and PbCl_2_ when interacting with nitrogen-functionalized carbonaceous surfaces. Additionally, a detailed analysis of the adsorption energies and critical geometric parameters is provided, as presented in Table 2.

A set of nine distinct adsorption configurations representing the interactions of Pb^0^, PbO, and PbCl_2_ with nitrogen-doped carbonaceous surfaces is seen in Figure 2. Notably, the active sites on the nitrogen-functionalized carbonaceous surface are found to be the unsaturated carbon atoms at the edges of the surface, as observed in Section 2.1 regarding pristine carbonaceous surfaces. These active sites serve as crucial loci for the adsorption of heavy metal species, and their interaction mechanisms are further elucidated in the context of nitrogen functionalization.

With the incorporation of nitrogen functional groups onto the carbonaceous surface, we observed significant changes in the adsorption behavior. The calculated adsorption energies for Pb^0^, PbO, and PbCl_2_ in the presence of nitrogen functional groups were found to range between −526.18 kJ/mol and −483.30 kJ/mol, −518.06 kJ/mol and −414.12 kJ/mol, and −346.21 kJ/mol and −288.31 kJ/mol, respectively, which are shown in Table 2. These values are notably lower than those obtained for adsorption on nitrogen-free carbonaceous surfaces, as indicated in Table 1. These results unequivocally indicate that the introduction of nitrogen functional groups onto the carbonaceous surface leads to a considerable enhancement in the adsorption capacity for Pb^0^, PbO, and PbCl_2_. This enhancement can be attributed to the presence of nitrogen moieties, which introduce additional interaction sites and offer a stronger affinity for the adsorbate species.

Furthermore, the MBO analysis for most of the chemical bonds formed during the adsorption process revealed consistent values close to 1.0, underscoring the predominantly single-bond formation in the adsorption of Pb^0^, PbO, and PbCl_2_ on nitrogen-functionalized carbonaceous surfaces. An intriguing exception was found in the bond C(7)—O(29) in structure 3-6, where an MBO value of 1.84 indicated the formation of a double bond. This observation aligns with empirical bond order principles and further reinforces the notion that MBO analyses accurately reflect the nature of chemical bonding during adsorption, distinguishing between single- and double-bond formations.

### 2.3. Electrostatic Potential Analysis

In chemical systems, a molecular electrostatic potential (ESP) analysis is considered as a dependable method for predicting nucleophilic and electrophilic sites. It can provide valuable insights into the distribution of electron density, thereby offering crucial information regarding the reactivity and interaction capabilities of molecules and surfaces. To extract more nuanced information about adsorption mechanisms, ESP analyses on van der Waals (vdW) surfaces have been extensively quantified to extract more nuanced information about adsorption mechanisms [38]. Building upon the established utility of ESP analyses, we draw inspiration from the work of Yang et al. [38], who effectively employed electrostatic potential calculations to unravel the adsorption mechanisms of aqueous Cd(II) on a MgO-modified palygorskite/biochar composite. This research highlighted the role of oxygen (O) atoms within the MgO group, which exhibited negative electrostatic potentials, signifying electrophilic activity. These insights into electrophilic sites shed light on the chemical interaction landscape of adsorbents and adsorbates, thereby enhancing our understanding of adsorption processes.

**Figure 3:**
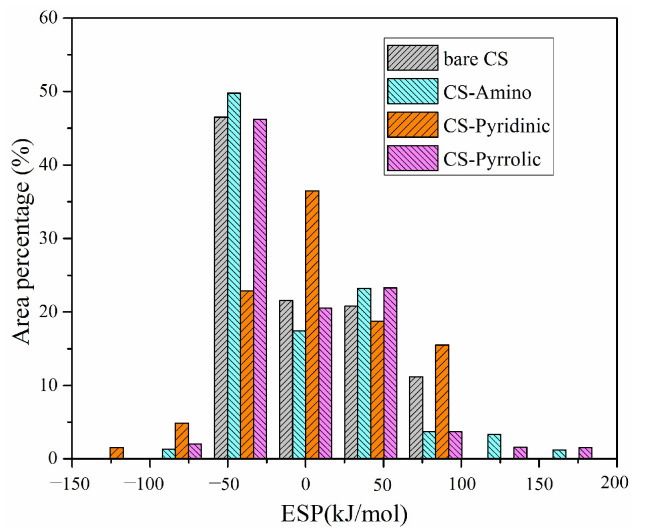
ESP distribution for different carbonaceous surfaces.

Therefore, ESP analyses on vdW surfaces for Pb^0^, PbO, and PbCl_2_ adsorption were further quantified to extract more information, and the corresponding percentages of different ESP values are presented in Figure 3. As seen from Figure 3, when examining the unmodified carbonaceous surfaces, the ESP distribution predominantly concentrates within the −50 kJ/mol to −100 kJ/mol interval. This range reflects the electrostatic characteristics of these pristine surfaces. However, upon the strategic incorporation of nitrogen functional groups onto the carbonaceous surfaces, a significant transformation in the electrostatic potential landscape is observed. Notably, the ESP distribution shifts towards higher absolute ESP values, indicating that the addition of nitrogen functional groups leads to an alteration in the electrostatic potential profile of the carbonaceous surfaces.

Strikingly, upon nitrogen functionalization, the ratio of electrostatic potential values in the larger absolute ESP range shows a substantial increase. Compared to the unmodified carbonaceous surface, the ESP values falling below −50 kJ/mol and above −100 kJ/mol increase by 5.87% with amino groups, 6.41% with pyridinic groups, and 6.30% with pyrrolic groups incorporated. These quantitative observations suggest that the presence of nitrogen functional groups exerts a notable influence on the electrostatic potential of the carbonaceous surface. Consequently, the propensity of the surface to interact with and adsorb Pb^0^, PbO, and PbCl_2_ is significantly enhanced.

**Figure 4:**
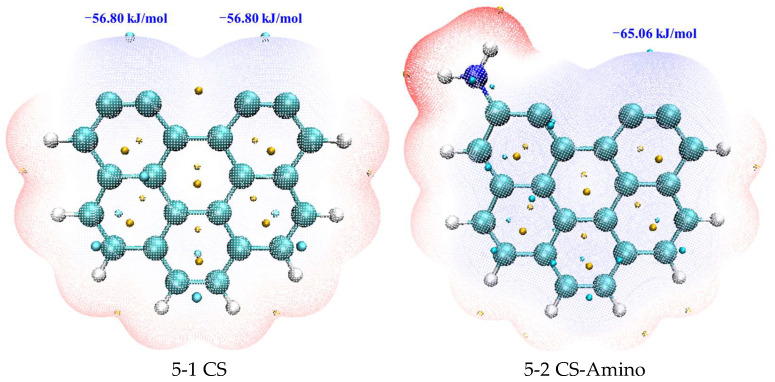

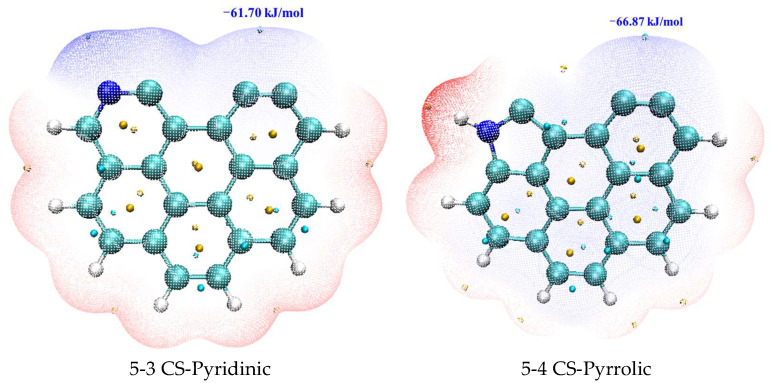
Electrostatic potential color-filled molecular surface map.

To further elucidate the mechanisms behind the enhanced adsorption capacity mediated by nitrogen groups, we quantitatively analyzed the molecular surface in Figure 4, gaining valuable insights into the changes in surface characteristics, topography, and interaction sites. In the electrostatic potential color-filled molecular surface map, red region means the positive ESP value, and blue region corresponds to the negative ESP value. In addition, the green ball is minimum point. This computational examination offers a comprehensive perspective on how nitrogen functionalization improves the adsorption capabilities of the carbonaceous surface. It elucidates the intricate adsorption processes and the subtle molecular interactions governing heavy metal species.

Additionally, the structural intricacies of a model denoted as structure 5-1 were closely examined, featuring a benzene ring with two unsaturated carbon atoms. The central region between these two unsaturated carbon atoms was identified as a critical point within the structure, characterized by a minimal ESP value of −56.80 kJ/mol. This unique feature allows for investigating how nitrogen functionalization alters the electrostatic landscape. Upon the strategic modification of the carbonaceous surface with nitrogen functional groups, pronounced alterations were observed in the electrostatic potential profiles of these active sites. Specifically, it was found that the ESP values at these active sites underwent changes, decreasing to −65.06 kJ/mol for the model incorporating amino functional groups (structure 5-2), −61.70 kJ/mol for the model with pyridinic functional groups (structure 5-3), and −66.87 kJ/mol for the model featuring pyrrolic functional groups (structure 5-4). These quantitative shifts demonstrate that nitrogen functionalization markedly modified the electrostatic characteristics of those sites.

The decreased ESP values at these active sites after nitrogen functionalization indicate an increased adsorption capacity of the carbonaceous surface, demonstrating that the incorporated nitrogen functional groups play a vital role in augmenting the electrostatic interactions between the surface and adsorbates. Consequently, the affinity of the carbonaceous material rose markedly for adsorbing Pb^0^, PbO, and PbCl_2_.

### 2.4. Density-of-States Analysis

A density-of-States (DOS) analysis quantifies the number of electronic states per unit energy level, thus offering critical insights into the distribution of electrons within a material as a function of energy. Thereby, it elucidates the electronic properties of materials and their interactions with adsorbed species. Chen et al. [39] utilized DOS analyses to explore heavy metal adsorption on a MnFe_2_O_4_@CAC hybrid adsorbent. In their research, it was revealed that the 4s orbitals of metal cations were occupied by the d electrons of the adsorbent, elucidating electron transfer and bonding mechanisms during the adsorption process.

**Figure 5:**
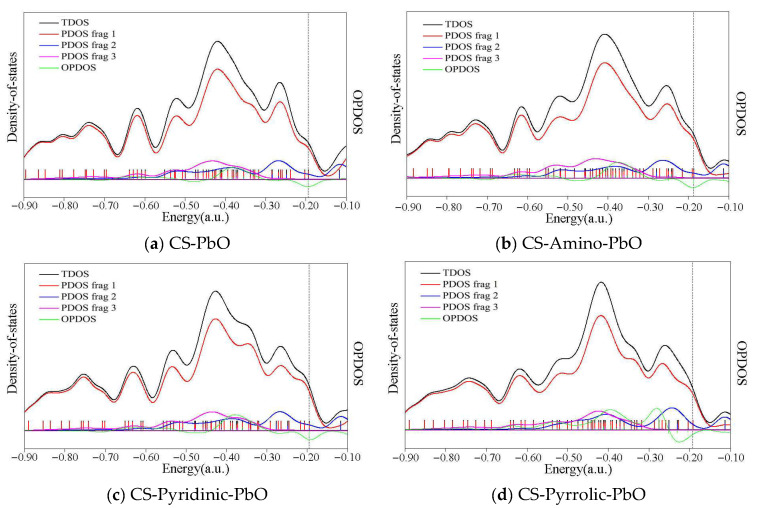
TDOS, PDOS, and OPDOS of PbO adsorption over bare and N-doped carbonaceous surfaces, frag.1 is C and N elements, frag.2 is PbO, and frag.3 is the H element of carbonaceous surfaces.

PbO adsorption was selected as a representative case for performing detailed DOS analyses across four distinct models in this investigation. The results are present in Figure 5, highlighting the total density-of-states (TDOS) in black and the overlap population density-of-states (OPDOS) in green. In addition, the partial density of states (PDOS) was examined for each optimized structure to gain insights into the electronic distribution governing gaseous PbO adsorption. The DOS energy range remains relatively consistent between the models in Figure 5. However, clear variations emerge in the density of states at different energy levels, which implies that nitrogen functional groups alter the electron distribution across the carbonaceous surface, thereby influencing the availability and capacity of active sites for adsorbing gaseous PbO.

Aligning with molecular orbital principles, an OPDOS analysis effectively visualizes the orbital composition and electron interactions. As emphasized in our prior work [21], positive OPDOS regions signify bonding interactions, while negative regions indicate antibonding. For instance, structure 2-2 in Figure 5a shows minimal positive areas in the OPDOS line, corresponding to the largest adsorption energy (−274.07 kJ/mol). In comparison, two pronounced peaks emerge around −0.4 and −0.3 a.u. in the OPDOS plot of structure 3-6 in Figure 5d, with larger positive regions that match its adsorption energy (−518.06 kJ/mol), demonstrating intensified electron overlap upon nitrogen functionalization, thereby enhancing the PbO interactions with the doped carbonaceous surface.

The DOS analysis revealed significant changes in the electron density distribution on the carbonaceous surface after nitrogen functionalization, implying a change in the availability of active sites for gaseous PbO adsorption. Larger positive areas emerge in the OPDOS plots of nitrogen-containing models, denoting intensified electron overlap between PbO and the surface. Therefore, greater electron sharing, enhanced by the incorporated nitrogen groups, strengthens the PbO interactions with the carbonaceous surface. Combined with the computed adsorption energies and Mayer bond orders, the DOS analysis consistently demonstrates that nitrogen functional groups bolster the carbonaceous surface’s adsorption capacity for lead species.

## 3. Models and Computational Methods

### 3.1. Carbonaceous Surfaces with Different Functional Groups

**Figure 6:**
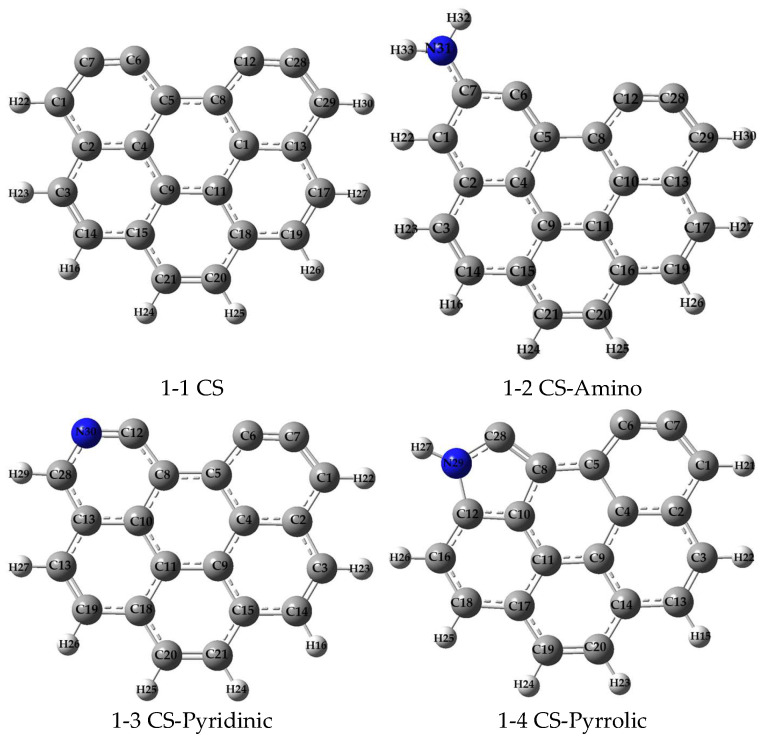
Models of carbonaceous surfaces (N, blue; H, light gray; and C, gray).

Currently, the quantum chemical method has been considered as the most accurate theoretical method for calculating molecular configuration and energy, of which density functional theory (DFT) has been widely employed due to its efficiency and accuracy [40,41]. In general, a simplified model that is reasonable can not only reduce the calculation workload, but also provide accurate results for complex systems. To simulate the structures of carbonaceous surfaces, armchair benzene rings were employed as the base models, which have been validated to reflect the properties of real carbonaceous materials [5,21]. Zou et al. demonstrated the feasibility of using armchair carbonaceous surface models by investigating the DFT calculations of arsenic adsorption [20]. Typical nitrogen functional groups were introduced onto the carbonaceous surfaces to examine the effects of the nitrogen modification of activated carbon on Pb adsorption. As illustrated in Figure 6, four types of carbonaceous surface models were constructed: (1) six armchair benzene rings (CS); (2) CS substituted with amino groups (CS-Amino); (3) CS substituted with pyridinic groups (CS-Pyridinic); and (4) CS substituted with pyrrolic groups (CS-Pyrrolic). This modeling approach allows for a systematic investigation of the mechanisms through which nitrogen functionalization enhances Pb adsorption capacity.

### 3.2. Calculation Method

All the calculations in this paper were conducted with the B3LYP method using the Gaussian 16 suite of programs. The def2svp basis set was used for the optimization of non-metallic elements (C, H, O, N, and Cl) [42], and the SDD basis set was applied to Pb elements. Frequency calculations were performed to check the imaginary frequency with the same theory level. In addition, the dispersion corrected (DFT-D3) was taken into account as a result of the weak interactions during the adsorption process [43,44]. Meanwhile, wavefunction analyses, including Mayer bond order, density-of-states, and electrostatic potential, were conducted with the help of the Multiwfn program. Fully unrestricted geometry optimizations were performed for each adsorbate system, which allowed all atoms to freely adjust their positions to reach the minimum energy configuration. Due to the adsorption of gas-phase lead molecules on the carbon surface, the total energy of its system would decrease. Therefore, the adsorption energy (*E*_ads_) can be calculated by the formula:

*E*_ads_ = *E*_(surface+molecule)_ − (*E*_surface_ + *E*_molecule_)

Here, *E*_(surface+molecule)_, *E*_surface_, and *E*_molecule_ are the total energies of the carbonaceous surface plus the molecule surface system, the carbonaceous surface, and the gas-phase molecule, respectively.

## 4. Conclusions

In this study, DFT calculations were performed to reveal the influence of nitrogen-containing functional groups, specifically focusing on amino, pyridine, and pyrrolic groups, on the adsorption of Pb^0^, PbO, and PbCl_2_ on carbonaceous surfaces. Various analytical techniques, including adsorption energy calculations, Mayer bond order analyses, electrostatic potential assessments, and density-of-states analyses, were employed to gain a comprehensive understanding of the adsorption processes.

It was found that robust interactions between the gaseous lead species and unmodified carbonaceous surface existed, as evidenced by the adsorption energies of −310.35 kJ/mol, −274.07 kJ/mol, and −260.96 kJ/mol for Pb^0^, PbO, and PbCl_2_, respectively. The introduction of nitrogen functional groups onto the carbonaceous surface demonstrated a consistent reduction in adsorption energy, signifying an augmented adsorption capacity for gaseous lead species on the modified surface. An in-depth analysis of the electrostatic potential and density-of-states unveiled the pivotal role of nitrogen functional groups in expanding the electrostatic potential distribution and fortifying electron overlap. The incorporated nitrogen moieties extended the charge distribution, therefore promoting lead adsorption. Additionally, a DOS analysis confirmed that nitrogen functional groups intensified the electron overlap between carbon atoms and lead, hence reinforcing the interactions between gaseous lead and the carbonaceous surface. These insights illuminate the mechanisms through which nitrogen functionalization augments adsorption interactions.

In summary, the comprehensive investigation underscores the substantial impact of nitrogen functional groups on the adsorption behavior of heavy metal lead species on carbonaceous surfaces. The modification of the carbonaceous material with these functional groups results in an improved adsorption capacity, and the associated changes in electrostatic potential and electron distribution further elucidate the underlying mechanisms governing these interactions. This research contributes to the understanding of the role of nitrogen-functionalized carbonaceous materials in environmental remediation and adsorption processes.

## Figures and Tables

**Table 1:** Adsorption energy, major bond lengths, and MBO for lead species adsorption on nitrogen-free carbonaceous surfaces.

Structure	E_ads_ (kJ/mol)	Bond	Bond Length (nm)	MBO
2-1 CS-Pb	−310.35	C(6)—Pb(31)	0.223	0.95
		C(7)—Pb(31)	0.226	0.91
2-2 CS-PbO	−274.07	C(6)—Pb(31)	0.225	0.91
		C(7)—O(32)	0.134	1.21
2-3 CS-PbCl_2_	−260.96	C(12)—Pb(31)	0.234	0.71
		C(28)—Cl(32)	0.177	0.95

**Table 2:** Adsorption energy, major bond lengths, and MBO for lead species adsorption on N-containing carbonaceous surfaces.

Structure	E_ads_ (kJ/mol)	Bond	Bond Length (nm)	MBO
3-1 CS-Amino-Pb	−526.18	C(5)—Pb(34)	0.229	0.87
		C(12)—Pb(34)	0.229	0.82
3-2 CS-Pyridinic-Pb	−483.30	C(6)—Pb(31)	0.232	0.77
		C(12)—Pb(31)	0.232	0.86
3-3 CS-Pyrrolic-Pb	−505.07	C(6)—Pb(30)	0.241	0.76
		C(28)—Pb(30)	0.232	0.90
3-4 CS-Amino-PbO	−414.12	C(6)—Pb(31)	0.224	0.93
		C(7)—O(32)	0.135	1.20
3-5 CS-Pyridinic-PbO	−426.41	C(6)—Pb(30)	0.224	0.91
		C(7)—O(31)	0.134	1.23
3-6 CS-Pyrrolic-PbO	−518.06	C(6)—Pb(28)	0.234	0.84
		C(7)—O(29)	0.125	1.84
3-7 CS-Amino-PbCl_2_	−322.99	C(12)—Pb(31)	0.236	0.69
		C(28)—Cl(32)	0.176	0.97
3-8 CS-Pyridinic-PbCl_2_	−288.31	C(11)—Pb(30)	0.234	0.70
		C(27)—Cl(31)	0.177	0.96
3-9 CS-Pyrrolic-PbCl_2_	−346.21	C(9)—Pb(28)	0.245	0.64
		C(25)—Cl(29)	0.176	0.96

## Data Availability

The data presented in this study are available on request from the corresponding author.
